# Exploring the perceived benefits of ethics education for laboratory professionals

**DOI:** 10.1007/s40889-022-00140-y

**Published:** 2022-03-08

**Authors:** Khojasta Talash, Chloe Anthias, Laura L. Machin

**Affiliations:** 1 Histopathology Department, Stockport NHS Foundation Trust, Manchester, UK; 2 Lancaster Medical School, Lancaster University, Lancaster, UK; 3 Anthony Nolan, London, UK; 4 The Royal Marsden NHS Foundation Trust, London, UK

**Keywords:** Ethics education, Ethics teaching, Medicine and ethics, Science ethics

## Abstract

Clinical laboratories face ethical challenges on a daily basis. The ethics training provided for clinical laboratory staff is variable, with some receiving no training. We aimed to explore the perceived benefits of ethics education for laboratory professionals. Ethics training was provided to approximately 60 laboratory professionals in a UK not-for-profit blood cancer organisation, with group discussions incorporated into the session. The session covered dominant ethical theories and principles, the defining moments in medical research ethics and the ethical aspects of laboratory practices. At the end of the session a short optional paper survey was distributed to the participants to obtain feedback on the training. The feedback was anonymous and thematically coded. Attendees reported to be more aware of the existence and importance of ‘everyday’ ethics in their workplace. Responses also showed that the training session had provided participants with an opportunity for ethical reflection in themselves and in discussion with their colleagues. Despite clinical laboratory professionals being faced with ethical challenges daily, there is comparatively little ethics education provided. Ethics training is believed to improve the ethical attitude of laboratory staff and help them when making decisions in their work. We have shown that ethics education is important for laboratory professionals to develop and retain ethical awareness, and ethical reflection. By gaining insight into the ethical aspects of their practices, laboratory professionals can apply this understanding when faced with making challenging decisions in their workplace, in order to act in the best interests of their patients.

## Introduction

Ethical issues arise daily in the clinical laboratory setting. Emerging infections, such as the current COVID-19 pandemic, mean laboratories are under more pressure to provide rapid results on a large scale, which can exacerbate the challenges that laboratory professionals face (Dubov et al. 2016). A minority of authors have contemplated the ethical aspects of laboratory work within Beauchamp and Childress’ (2001) four ethical principles (see Appendix Table 3) and occasionally by drawing upon dominant ethical theories such as virtue ethics (Stempsey 1989). Others have considered the various ethical challenges laboratory staff can face at each stage of the laboratory process (see Appendix Table 4) (for an in-depth discussion of the ethical challenges presented at different phases of the laboratory process see Nyrhinen and Leino-Kilpi (2000)).

To support laboratory professionals facing these challenges, various international and national ethical codes have been developed by associations, societies, and governments (Government Office for Science 2007; Institute of Biomedical Science 2014; International Federation of Biomedical Laboratory Science 2010; Statland 2007; The Association for Clinical Biochemistry and Laboratory Medicine 2013), stating how these individuals should act. They include maintaining good standards and keeping up to date with codes of practice. However, there is evidence that individuals have different levels of understanding of ethical concepts (Mabrouk 2013). Moreover, a concern has previously been expressed over some senior laboratory professionals’ attitudes towards communicating with patients (Hernandez 2011). Together, this suggests that codes of conduct are not sufficient on their own to promote ethical behaviour, and additional support is required to foster ethical awareness and reflection within the clinical laboratory setting.

As advancements in technology continue at a rapid pace, having the ability and opportunity to reflect on the ethical aspects of their practices is a necessity for laboratory staff (Stempsey 1989), particularly since information technology is becoming an increasingly key component in their workplace (Jones et al. 2014). Furthermore, it is important for laboratory staff to be aware of the ethical issues within their workplace since around 70% of medical diagnoses require laboratory tests (Lord Carter of Coles 2008), and therefore clinical medicine relies heavily on the work carried out in the laboratory. Many of these results are expected to be available after only a short period of time, for example suspected cancer cases that fall into the four-week wait category. The high turnover rate means laboratory workers are under pressure to provide results quickly, however care must be taken to ensure the correct results are released, to avoid patient harm and emotional distress (Nyrhinen and Leino-Kilpi 2000).

Medics, ethicists, scientists and psychologists have all proposed that laboratory staff should develop their ethical reasoning ability in order to adopt a systematic approach to their work (Wijeratne and Benatar 2010), and thereby prioritise the needs of patients. Whilst previous studies suggest that some laboratory staff may be able to apply ethical reasoning better with increased experience over the years (Schlaefli et al. 1985), other studies indicate that ethics training is vital to build skills to allow individuals of all ages to recognise ethical challenges (Clarkeburn et al. 2002). Ethics training has been shown to provide fast positive results (Smith et al. 2007) and to improve ethical attitudes of clinical laboratory staff (Khalajzadeh et al. 2019). Despite the positive reviews of ethics training, previous studies have highlighted an inadequacy in the ethics training provided to laboratory workers (Bruns et al. 2015; Domen 2002), which starts at the early stages of a life sciences career (Healey 2015). This may be symptomatic of the ethical challenges that laboratory professionals face being under-recognised (Domen 2002), or simply that the voice of those working in laboratories is missing from many of the ethical debates related to laboratory work. It is clear therefore that the ethical training needs of laboratory professionals have been overlooked and neglected.

This paper reports the perceived need for ethics training amongst clinical laboratory professionals. Drawing upon the views of laboratory staff who received ethics training, we focus on the ethical challenges that staff face daily in the laboratory, as identified by the participants, so called ‘every day ethics’. We also explore the perceived benefits of receiving ethics training, namely an increase in the ability to identify ethical issues in a given situation which we refer to in this article as ethical sensitivity, as well as providing a time for individuals to reflect on the ethics of their practice. We conclude that training for laboratory workers that enhances their ethical awareness and encourages ethical reflection is vital. We also provide an outline of the ethics training delivered and share materials that may be used in future sessions, in order to encourage others to provide ethics training for laboratory staff.

## Methods

Ethics training was delivered to approximately 60 laboratory staff at a UK not-for-profit blood cancer organisation in July 2019 and February 2020. The session lasted 90 min and was delivered on multiple occasions with approximately 15 members attending each time. The training was interactive, involved small group work and large group discussion, and covered ethical theories and principles, the defining moments in medical research ethics and the role of ethics in the laboratory (see Table 1).

**Table 1:** An overview of the ethics training session delivered to laboratory staff

Starter Task: Explore participants’ perceptions surrounding, and understanding of, ‘ethics’	What does ‘ethics’ mean to you? What role or function do they play? Why do they matter? Who do they serve? Do they differ across professions or are they universal? Are they static or evolving? Are they the same as regulations and laws?
Discuss common ethical theories and principles	For example, discuss **deontology** (duty based theory), **virtue ethics** (character trait development), and **consequentialism** (the right act is the one that promotes the best consequences) Discuss the four principles of ethics: Beneficence, non-maleficence, justice, and autonomy Explore how the participants feel the theories and principles relate (or not) to their practices, roles and responsibilities
Reflect on the defining moments in the development of ‘ethics’ to understand the emergence of some common ethical considerations	For example: Nuremberg Trials; the Declaration of Helsinki Alder Hay organ retention inquiry; the Human Tissue Act and Authority Henrietta Lacks; patient consent for involvement in research, providing compensation for involvement in research
Consider current understanding relating to laboratory ethics	Provide an overview of the published literature on the topic including journal articles, research studies, and international codes of conduct Include identified ethical principles and values which are specific to laboratory work i.e. integrity, autonomy, confidentiality, social responsibility, maximise benefits, minimise harms, courage
Discuss and develop participants’ laboratory ethics	Where are the ‘ethics’ in your role with reference to equipment, technology, clothing, computer, people, committees, paperwork, environment, spaces? What are your ethical principles that underpin your work? When do these principles arise? Matter? Conflict? Do some take priority—when? Why?

Participants had the opportunity to ask questions and share their views with the group throughout the session. At the end of the discussion participants were asked to complete a short optional paper survey to provide their opinions on the training delivered. The survey consisted of six questions including what participants found most challenging and beneficial about the training, and how the training supported and related to participants’ current role and work.

In total 43 attendees completed the optional survey. The survey results were collated and qualitatively analysed, identifying recurring themes in the responses (Boyatzis 1998). As the survey was anonymous, demographic data was not obtained from the respondents. Permission was sought from participants and the senior leadership team to use participants' survey responses for research purposes. Ethics approval was obtained from the Faculty of Health and Medicine Research Ethics Committee, at Lancaster University.

## Results

Responses to all questions were gathered together and coded into themes, with three prominent emerging themes—ethical sensitivity, everyday ethics, and ethical reflection—with some overlap between the themes.

### Increased ethical sensitivity

Some respondents perceived the training to help them to develop an awareness of ethical concepts and enabled them to make connections between the ethical concepts and their duties in the laboratory. For example, when asked how the training related to and supported the individuals in their work, respondents claimed:

‘Understanding more what ethics is and the impacts it may have in my job’.

‘Bringing ethics to the front of my mind and actively linking ethics to my role’.

‘It made me think about how ethics underpins my job’.

Together these quotes illustrate that having the training helped staff appreciate the ethical aspects of their work. By becoming more ethically sensitive attendees were able to realise why certain considerations and decisions are important in their roles. The following quotes are in response to how the training supported laboratory staff in their work:

‘Reminding me of the foundation of some of my working patterns and why I make the decisions I do’.

‘Good to remember why we behave how we do following our ethical principles’.

‘Allowed us to think about how ethics influences our work - easily forgotten’.

‘Reminds us why we do things in certain ways’.

Furthermore, when explaining how the training related to their work, other respondents said:

‘In order to be able to deliver the right outcome by following what is good and right and not what suits me’.

‘Carrying out routine work day to day can make you lose sight of the end role - it was good to see that I do always think about the right thing’.

These responses show that by developing ethical sensitivity in the attendees, the training helped act as a reminder for the laboratory staff of the purpose of their work. In essence, the training allowed attendees to increase their understanding and awareness of how ethics informs and influences their work. By doing so, the training helped reinforce the importance of the work laboratory staff do and why acting ethically is so vital.

### Recognising the ‘everyday’ ethics

Participants were more aware of the general ethical aspects of their work, and significantly were also able to recognise the ways in which ethics affects their decision making in their common and recurring practices. For some attendees the most beneficial aspect of the training was having the chance to consider the role ethics plays in their daily duties, as the following quotes illustrate:

‘Having the possibility to summarise in my mind the ethics values and how I try everyday to uphold them’.

‘Understanding how I use ethics every day without realising’.

‘Being able to relate the topics we spoke about back to the work I do every day’.

These responses illustrate how the ethics training encouraged the participants to identify the ethical challenges they face in their day to day work, something that they may not have been aware of previously. When asked how the training supported the individuals in their work, three participants replied:

‘Brings more awareness to activities you deem are ‘normal’ to do every day’.

‘How ethics are a part of everyday working life’.

‘Getting me to really think about how ethics affects everything I do’.

By increasing ethical sensitivity, it appears attendees were able to evaluate what they perceived as routine daily work and thereby appreciate how ethics has shaped what they do and why. Similarly, some laboratory staff stated that they felt the training provided them with an opportunity to recapture the importance of ethics in their daily duties:

‘It has reminded me about what I feel is important to me in my everyday work’.

‘A useful reminder of how ethics is a part of my day to day job’.

Some laboratory professionals identified particular activities in their day to day work that they perceived the ethics training applied to and promoted, namely ‘work prioritisation’, ‘working within quality management’ and ‘working closely with patient information’. Furthermore, when replying to how the training related to their work, one participant described an appreciation for the way in which ethics impacts daily decision making in relation to coworkers and patients:

‘It affects everyday work even in minor ways as we are always making ethical decisions that affect our colleagues or patients’.

It appeared therefore that the ethics training encouraged laboratory staff to recognise the ways in which ethics played a key role in many of their day to day activities.

### A time for reflection

A number of participants felt the training session provided a much needed opportunity for them to reflect on their work. Staff were reminded of the vital nature of their work and how their role contributes to patient care. In response to how the training supported the individuals in their work, some laboratory staff reported:

‘Puts into perspective our role in the link of healthcare between medical professional and patient/donor’.

‘As a reminder of what we should do for the wellbeing of others’.

The value of having time and space to reflect on their working practices was highlighted by several participants as the most beneficial aspect of the training:

‘It has helped me remember what I came into work for, as it can be easy to forget. I love conversations that require deep thought’.

‘It has helped me sit back and really think about the impact that my job has’.

‘Been really fascinating. Time to think and focus on what we do on autopilot’.

Participants also perceived they benefited from being able to discuss their views with other members of the group. For example, one respondent stated that for them the most beneficial aspect of the training was, ‘The opportunity of understanding other people’s vision and their principles’. The training therefore appeared to facilitate laboratory staff to reflect on their personal views, as well as the ethical perspectives of their colleagues. By fostering conversations that may not otherwise have taken place, the training highlighted the importance of providing laboratory professionals with a space to discuss and reflect upon the ethical aspects to their work.

## Discussion

Clinical laboratory ethics has so far been somewhat neglected from being addressed, despite laboratory professionals frequently facing ethical challenges (Domen 2002). Although previous studies have advocated the use of ethics training (Clarkeburn et al. 2002; Khalajzadeh et al. 2019; Smith et al. 2007), such training opportunities for clinical laboratory staff have been limited (Bruns et al. 2015), with a varied exposure to ethics training even for undergraduate life sciences students (Healey 2015).

In this paper, we have shared resources from our structured facilitated workshop, which can be used and adapted by departments and organisations to provide clinical laboratory teams with ethics training in their workplaces. Our results emphasise the benefits as perceived by the participants and therefore reinforce the importance of providing ethics training to laboratory staff. Our findings also show that laboratory professionals are willing to engage in ethics education, and applying such learning to their everyday practices. It is apparent therefore that there is a demand for ethics training amongst laboratory workers, which has largely been unmet so far.

Our results have shown that increasing the understanding of the role of ethics in the work of laboratory professionals helps to remind them of the importance of the routine daily work performed in the clinical laboratory. As acknowledged in previous research, our results show that ethics training helps promote ethical sensitivity (Clarkeburn et al. 2002) as well as ethical reflection, thus equipping laboratory professionals with the skills to be able to perform their daily duties in a structured and organised manner, putting patient needs first (Wijeratne and Benatar 2010). This will allow clinical laboratory departments to work more efficiently and thus be able to meet the increasing time pressured demands for releasing optimum diagnostic results. This is vital in a time when technology is so frequently used in the laboratory (Stempsey 1989), when diagnostic medicine relies so heavily on laboratory services (Lord Carter of Coles 2008), and even more so in the face of the current coronavirus global pandemic. Furthermore, a laboratory team that can weigh up the benefits and drawbacks of different decisions in their department, for example when buying new laboratory equipment, can help save costs and ensure resource allocation is just, the importance of which has been previously highlighted (Wijeratne and Benatar 2010). Finally, this will feed into helping patients receive the best care by providing clinicians with the appropriate results to ensure the next steps of a patient’s treatment are informed and well planned.

Our study has some limitations; a small cohort of laboratory professionals were surveyed, since the training sessions were delivered to employees of a single organisation. Furthermore, not everyone who attended the training session completed the survey; therefore there may be a bias such that those more enthusiastic about ethics training were more likely to complete the survey. However, our results identify recurring themes amongst the majority of respondents relating to the perceived benefits of ethics training, which cannot be ignored.

Future research could explore the use of other initiatives and how successful they prove to be in promoting ethical awareness, reflection and sensitivity amongst clinical laboratory professionals. Such initiatives may include running relevant journal clubs, and discussing case studies (see Table 2). Pre- and post-training quizzes could be used to assess how much the participants learned during their training. These initiatives are proposed as they each encourage and promote the voice of laboratory professionals, and in turn have the potential to inform and influence the ongoing development of the specialty ‘laboratory ethics’.

**Table 2:** Suggested case studies for laboratory ethics education sessions

Case Study	Discussion Prompts
Handling patient information: An interesting case has arrived in the laboratory, which has features that are very rarely seen in clinical practice. One of your senior colleagues would like to take a photograph of the sample for teaching purposes and publication; however you notice that the consent form has not been signed by the patient for use of material for research or teaching purposes	What potential actions can you take? What factors may influence your actions? What are the considerations that need to be made? Would the patient come to harm if their tissue was used for teaching/publication in this case? If no, does that make it okay to use?
Performing the tests: You have been processing samples continuously over the last two hours to try to clear the backlog of specimens arriving into your department. As you pick up the next specimen you notice a piece of a specimen that has been left behind on your workspace. You are not sure which case it belongs to, since you have processed many samples already	What potential actions can you take? What factors may influence your actions? What are the considerations that need to be made? What action would be in the best interests of the patients?
Reporting the results: Whilst authorising reports of test results so that they can be released to the requesting medical team, you come across a particular case where the tests requested by the clinicians do not match up with the clinical questions they are trying to answer – i.e. the tests performed are inappropriate and will not answer the clinical question. You submit the report of the results, which were all normal. Are there any other steps you should take?	What is your duty/role in this scenario? What are the potential actions you can take? What factors may influence your actions? What actions will be in the best interests of the patient?

## Conclusion

In conclusion, ethics training is an important tool that should be used in clinical laboratory departments to help promote ethical sensitivity and recognition of the ‘everyday’ ethics. The training also offers a space for staff to reflect on their work and thus provide a reminder of the impact of the decisions they make daily on the care that patients receive. By having laboratory professionals that are aware of the ethical aspects of their work, we can increase the determination of staff to do what is right and is best for the patient, and thus continually aspire to achieve and maintain excellence within the workplace.

## Appendix

**Table 3:** The four ethical principles applied to laboratory medicine

Baron (1993)	*• Autonomy*—checking patient’s informed consent, maintaining confidentiality *• Beneficence*—seeking expert advice on complex cases, informing clinicians immediately of unexpected results *• Non-maleficence*—avoiding mistakes, writing good reports, advising clinicians of the limitations of certain tests *• Justice*—aiming for fair resource allocation
Sobel (1999)	*• Autonomy*—considering each individual’s opinions and choices *• Beneficence (and non-maleficence)*—maximising benefits, minimising harm *• Justice*—ensuring research subjects are among the beneficiaries of the research
Nyrhinen and Leino-Kilpi (2000)	*• Autonomy*—patient counselling, checking informed consent, privacy *• Beneficence*—risk factor analyses of genetic information *• Non-maleficence*—obtaining optimal benefit from a procedure *• Justice*—justification of genetic screening
Burnett et al. (2007)	*• Autonomy*—checking patient’s informed consent, maintaining confidentiality, recognising and respecting conflicts between individual self-determination and cultural/religious beliefs *• Beneficence*—contributing to greater good of individuals in society, balancing societal needs against individual benefit *• Non-maleficence*—protecting patient privacy, minimising impact on individual’s background and beliefs *• Justice*—respect individual’s values, ensuring equal access to healthcare resources
Wijeratne and Benatar (2010)	*• Autonomy*—checking patient’s informed consent, maintaining confidentiality *• Beneficence and non-maleficence*—awareness of how medical decisions can carry risks of harm as well as benefits to the patient *• Justice*—balancing individual good with public good when allocating resources
Bhagwat and Pai (2020)	*• Autonomy*—checking patient’s informed consent, confidentiality. Protecting patient information *• Beneficence*—providing good written reports, offering medical advice beyond just diagnosis, where appropriate *• Non-maleficence*—disclosing errors, concept of over-diagnosis *• Justice*—fair and equal allocation of resources

**Table 4:** Ethical challenges in laboratory medicine

Handling patient information	Checking for patient’s informed consent, ensuring results are maintained confidential unless disclosure is authorised. Ensuring that there is adequate privacy during reception and sampling (Arora and Arora 2007) Safeguarding of patient information, ensuring only authorised individuals can alter results (World Health Organisation 1999)
Performing the tests	Carrying out work with high level of competence, maintaining patient’s best interests. Informing clinicians where a test is going to produce unreliable results, rather than performing the test (World Health Organisation 1999)
Reporting results	Challenges of how much information to include in the report, e.g. avoiding potentially harmful over-reporting (McGuire et al. 2013; World Health Organisation 1999)
Error reporting	Challenges of identification and reporting of errors, e.g. considering whether the patient will understand the meaning of the error, the challenges of reporting someone else’s errors (Perkins 2016)
Research	Informed consent in the context of research (Domen 2002; McQueen 1998) Data storage, use of biological materials for secondary studies (McQueen 1998)
